# Low parental income level is associated with pediatric COVID-19 vaccine hesitancy in the San Francisco Bay area

**DOI:** 10.1186/s12889-025-22132-5

**Published:** 2025-03-07

**Authors:** Overbeck Christian Takou Mbah, Sara H. Goodman, Yvonne Maldonado, Jennifer Bollyky

**Affiliations:** 1 Stanford University School of Medicine, Stanford, CA USA; 2 Department of Pediatrics, Division of Infectious Diseases, Stanford University School of Medicine, Stanford, CA USA; 3 Department of Epidemiology and Population Health, Stanford University School of Medicine, Stanford, CA USA; 4 Department of Medicine, Division of Primary Care and Population Health, Stanford School of Medicine, Stanford, CA USA

**Keywords:** COVID-19 vaccine hesitancy, Vaccine acceptance, Vaccine uptake, Vaccine hesitancy scale (VHS), Income, Socioeconomic status, San Francisco Bay area, Parental attitudes, Pediatric vaccination, Child immunization

## Abstract

**Objective:**

To characterize the differences between COVID-19 vaccine-hesitant and vaccine-accepting parents in the Bay Area.

**Methods:**

We analyzed a cross-sectional survey of 251 parents of children (< 18 years) from six San Francisco Bay Area counties in November of 2022. We adapted WHO’s Vaccine Hesitancy Scale (VHS) into a 9-item Likert scale measuring COVID-19 vaccine hesitancy. We assigned a VHS score to each participant, with lower VHS scores indicating higher vaccine hesitancy. We performed multiple linear regression analyses with VHS scores and children’s vaccination status as outcomes and demographic factors as predictors.

**Results:**

Parents of children who had received at least one dose of the COVID-19 vaccine had a 17.1% higher VHS score compared to parents of children who had not received any dose (*p* < 0.01). Additionally, parents with annual income <$50,000 had a 9.6% lower VHS score compared to parents with income > $150,000 (*p* < 0.01), indicating higher vaccine hesitancy in lower-income parents.

**Conclusions:**

Low-income parents appear to be more vaccine-hesitant than high-income parents in the Bay Area. Future public health interventions should focus on lower-income parents to improve vaccine uptake.

**Supplementary Information:**

The online version contains supplementary material available at 10.1186/s12889-025-22132-5.

## Introduction

COVID-19 vaccination rates in children in the US and California are lower than for adults. As of May 3, 2023, the Centers for Disease Control and Prevention (CDC) reports that national rates of completion of primary series of vaccination are 59% in ages 12–17, 32% in ages 5–11, and 13% for the first dose in 6 months-4-year-olds [1]. The state of California similarly reports suboptimal vaccine rates. As of July 6, 2023, the rates of completion of the primary series of vaccination in California are 67.3% in ages 12–17, 37.8% in ages 5–11, and 8.6% in ages 5 and younger [2]. While vaccination rates are lower in the pediatric population, children remain facilitators of SARS-CoV-2 transmission [3]. To better understand the causes of these low vaccination rates among children and inform public health interventions, we investigated factors contributing to pediatric COVID-19 vaccine hesitancy among parents in the Bay Area, California. Of the multiple factors that were explored, this study focused on sociodemographic influences.

National studies have found that reasons for pediatric COVID-19 vaccine hesitancy are numerous: the lack of confidence in the safety and effectiveness of the vaccine, trust in the government, and perceptions that children are not susceptible to the disease [4–8]. Other studies have found an association between pediatric vaccine hesitancy and demographic factors [4, 9–11]. However, these studies were conducted before October 29, 2021, and thus prior to the earliest authorization of any COVID-19 vaccine in children 5–11 years of age. Furthermore, many of these studies do not rely on the Vaccine Hesitancy Scale (VHS), a survey tool originally designed by the WHO’s Strategic Advisory Group of Experts on Immunization (SAGE) to standardize the identification of vaccine hesitant groups [12, 13]. It offers a shorter and more comprehensive set of questions compared to the various tools used previously [13]. In this study, we evaluate vaccine hesitancy among parents of San Francisco Bay Area children (age < 18). We hypothesized that vaccine hesitancy in this population is multifactorial and includes demographic influences. To investigate this hypothesis, we conducted a cross-sectional survey using the VHS [14].

The WHO’s SAGE defines vaccine hesitancy as any delay in acceptance or refusal of vaccination despite the availability of vaccination services [15]. In 2015, the WHO’s SAGE developed the Vaccine Hesitancy Scale (VHS), a 10-item scale that allows the identification of vaccine-hesitant individuals [13]. It has since been validated in various diseases and socioeconomic settings [14, 16–18]. In this study, we adapted the VHS of Helmkamp et al. [14] with improved psychometric properties for childhood vaccines to identify vaccine-hesitant individuals in our Bay Area population. We then characterized the differences between vaccine-hesitant and vaccine-accepting individuals in demographic characteristics (such as income, race, gender, parental education level, and geographic location by county) and vaccine-hesitancy factors defined by the WHO’s SAGE in their vaccine hesitancy matrix.

## Methods

### Study design and sample

Our study was a cross-sectional survey administered between November 9, 2022, and December 3, 2022, nested within the TrackCOVID study, a longitudinal cohort study of the prevalence and incidence of SARS-CoV-2 infection in a representative population in the Bay Area. The TrackCOVID study included 3935 adults (18 years or older) from randomly selected households from the following six counties: Santa Clara, San Mateo, Alameda, Marin, Contra Costa, and San Francisco [19]. Inclusion criteria in our cross-sectional study were as follows: participants had to be adult parents or guardians of at least one child of age < 18 years, and residents of the six Bay Area counties during the duration of the cross-sectional study.

**Fig. 1:**
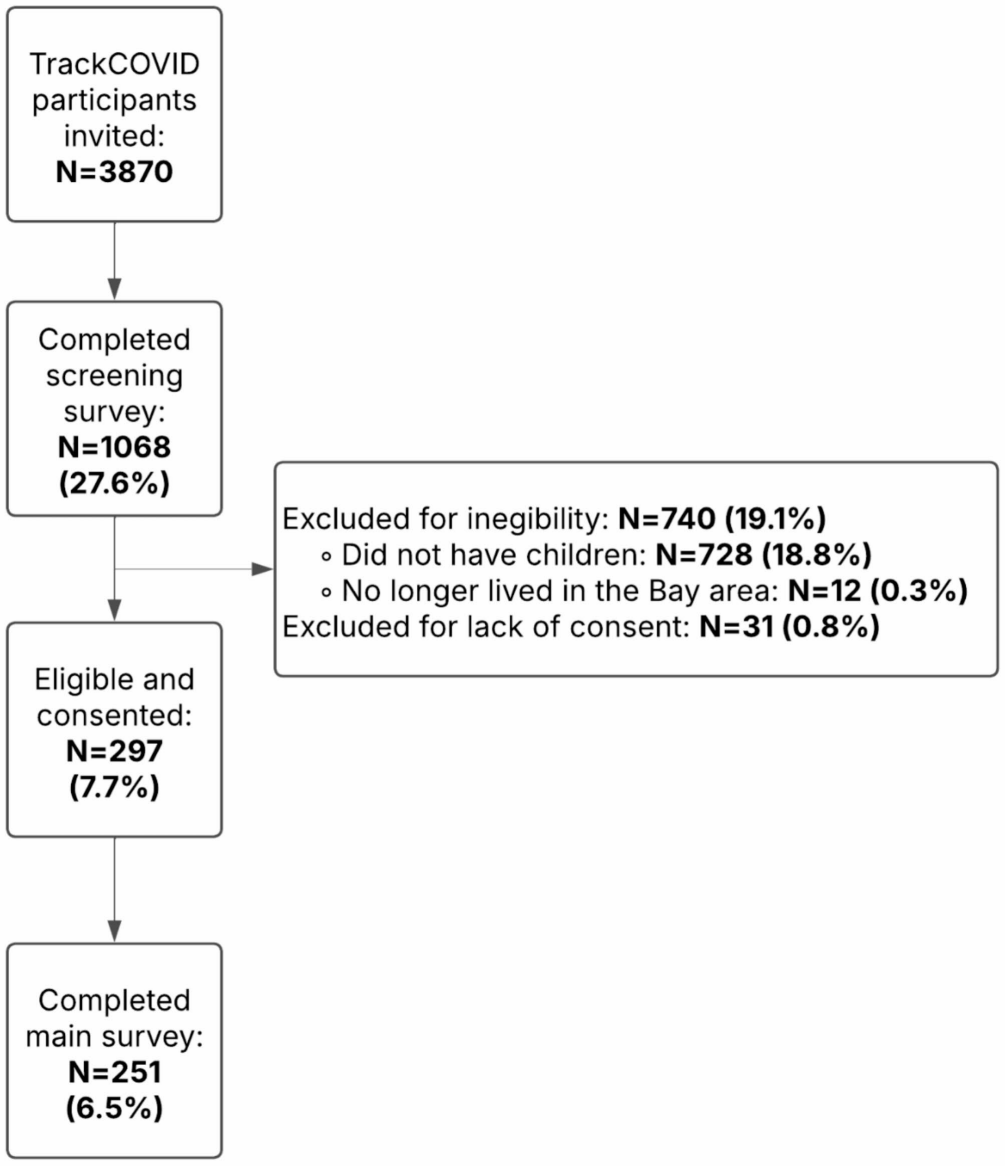
Flowchart of study participants

From the TrackCOVID study sample, 3870 individuals had indicated an interest in participating in future research and were invited to be study participants. 1068 individuals (27.6% of TrackCOVID participants) completed the preliminary screening survey. 297 individuals (7.7%) were eligible and consented to the study. A total of 251 participants (6.5%) completed the main study survey (Fig. 1).

### Survey development

Our main study survey was adapted from Helmkamp et al.’s VHS [14]. This is a validated 9-item Likert scale with improved psychometric properties for childhood, influenza and HPV vaccines. The wording of the VHS was adapted by our team to measure hesitance toward COVID-19 vaccines. The dimensions of the Likert scale included “Strongly disagree”, “Disagree”, “Agree”, and “Strongly agree”. The 9 items of our COVID-19 VHS (CoV-VHS) completed by participants are shown in the appendix.

In addition to the CoV-VHS, participants answered questions about sociodemographic factors including their vaccination status, prior positive COVID-19 test status, number of children in the household, the age of the index child, and the vaccination status of the index child. Participants with more than one child were asked to refer to the child with the closest birthday on the calendar as the index child. Other participants’ demographic characteristics were extracted from the longitudinal TrackCOVID database for analysis. Our research was approved by the Stanford University Institutional Review Board protocol number 65845 - Assessing Parents’ Hesitancy in Vaccinating Adolescents and Children against COVID-19.

### Scoring and statistical analysis

We assigned scores of 1, 2, 3, and 4 to the dimensions of the Likert scale, “Strongly disagree”, “Disagree”, “Agree”, and “Strongly agree”, respectively. We reverse-coded items b, e, and h from the CoV-VHS (see appendix) so that a higher score indicates less hesitancy (higher vaccine acceptance) on all items. We summed the scores across all items by participants, creating an individual VHS score. We normalized the data on a 0-100 scale. Our scoring method differs from that of Helmkamp et al.’s in that in our case, higher VHS scores indicate less hesitancy (higher vaccine acceptance), and a scale of 0-100 was used. Both of these changes were made to improve clinical interpretability. We performed multiple linear regression analyses assessing the relationship between VHS scores and children’s vaccination status (defined as having received the first dose or not), and factors contributing to vaccine hesitancy including demographics. P value < 0.05 was considered significant with 95% confidence intervals. All statistical analyses were performed using Stata, version 18.0 [20].

## Results

This study included 251 parents from six Bay Area counties, with Santa Clara and Alameda having the highest proportions of participants, 63 (25.1%) and 60 (23.9%) respectively, and Marin County having the lowest proportion of participants, 22 (8.8%, Table 1). Most parents in our sample had an annual household income of $150,000 or greater (132, 52.6%), but there is a nonnegligible minority of parents with an annual household income lower than $50,000 (16, 6.4%). In terms of education level, our sample was primarily comprised of parents with bachelor’s and master’s degrees, 106 (42.2%) and 77 (30.7%) respectively. Most participants in our study were White and Asian, 145 (57.8%) and 86 (34.3%), with only 12 (4.8%) Black or African American parents, and fewer Indigenous American parents (4, 1.6%) and Native Hawaiian or Pacific Islander parents (3, 1.2%).

**Table 1:** Description of participants’ demographics

Characteristics	*N*	(%)
**Gender**		
Male	101	12.7
Female	150	100.0
**Hispanic origin**		
Hispanic	32	12.7
Non-Hispanic	219	87.3
**Race**		
White	145	57.8
Black or African American	12	4.8
American Indian, Alaska Native, or Other Indigenous	4	1.6
Asian	86	34.3
Native Hawaiian or Other Pacific Islander	3	1.2
Other race	12	4.8
**Age Group**		
20–29	3	1.2
30–39	61	24.3
40–49	107	42.63
50–59	70	27.89
60–69	9	3.59
70–79	1	0.4
**Education level**		
Less than high school	2	0.8
High school diploma or equivalency (GED)	16	6.4
Associate degree (junior college)	15	6.0
Bachelors degree	106	42.2
Masters degree	77	30.7
Doctorate	16	6.4
Professional (MD, JD, DDS, etc.)	19	7.6
Other	0	0.0
**Annual Household Income**		
Less than $50,000	16	6.4
$50,000 through $100,000	32	12.7
$100,000 through $150,000	40	15.9
$150,000 or more	132	52.6
Decline to respond/Don’t know	27	10.8
**County**		
Alameda	60	23.9
Contra Costa	31	12.4
Marin	22	8.8
San Francisco	30	12.0
San Mateo	45	17.9
Santa Clara	63	25.1
**Prior Positive Status**		
No	100	39.8
Yes	151	60.2
**COVID-19 Vaccination Status of Parent**		
1st dose		
Received	247	98.4
Did not receive	1	0.4
2nd dose		
Received	227	90.4
Did not receive	0	0.0
1st booster		
Received	224	89.2
Did not receive	17	6.8
2nd booster		
Received	149	59.4
Did not receive	69	27.5
3rd booster		
Received	53	21.1
Did not receive	69	27.5
**Number of Children in the Household**		
1	134	53.4
2	95	37.8
3	18	7.2
4	3	1.2
More than 10	1	0.4
**Age of Index Child**		
< 6 months	4	1.6
6 months − 4 years	66	26.3
5 years − 11 years	86	34.3
12 years − 17 years	97	38.6
**COVID-19 Vaccination Status of Index Child**		
1st dose		
Received	219	87.3
Did not receive	32	12.7
2nd dose		
Received	207	82.5
Did not receive	5	2.0
1st booster		
Received	136	54.2
Did not receive	64	25.5

In our study, parents reported the vaccination status of a single randomly selected index child. We found that 219 (87.3%) children received at least 1 dose of the COVID-19 vaccine, with a substantial 32 (12.7%) who did not receive the first dose at the time of the survey. Interestingly, nearly all parents in our sample (247, 98.4%) had received at least one dose of COVID-19 vaccine.

We performed multiple regression analyses with the VHS score as a continuous the dependent variable and demographics as the independent variables as reported in Table 2. We found a positive association between the vaccination status of the child and the VHS score of the parent: parents of children who had received at least one dose of the COVID-19 vaccine had a statistically significant 17.1% higher VHS score compared to parents of children who had not received any dose, indicating higher vaccine acceptance in parents of vaccinated children. Importantly, VHS scores were also positively associated with parents’ reported annual household income: parents with an annual household income lower than $50,000 had a statistically significant 9.6% lower VHS score compared to parents with an annual income of $150,000 or greater, indicating lower vaccine acceptance (or higher vaccine hesitancy) among the lower-income parents. Notably, no education level or race was associated with significant differences in VHS scores in our sample. We also performed a descriptive analysis of the attitudes of parents toward vaccination in terms of contextual, individual/group and vaccine-specific influences. We observed that 54.7% of parents with parents with higher vaccine hesitancy (lower VHS score) agreed or strongly agreed with the statement “I believe the vaccine is not needed for my child”, compared with 1.9% of parents with less vaccine hesitancy. We also observed that 57.1% of parents with higher vaccine hesitancy disagreed or strongly disagreed with the statement “I trust the government is making decisions in my best interest with respect to the COVID-19 vaccine” compared with 11.5% of parents with less vaccine hesitancy (supplemental Fig. 1).

**Table 2:** Multiple linear regression on VHS score

COVID-19 Vaccination Status of Index Child	VHS Score (95% CI)	*P* value
Non-vaccinated	Reference	
Vaccinated with at least 1 dose	17.06 (13.22–20.89)	*p* < 0.01
**Age in Years**	-0.0223 (-0.183–0.138)	
**Race**		
White	Reference	
Black or African American	-1.572 (-8.120–4.976)	
American Indian, Alaska Native, or Other Indigenous	-3.356 (-14.96–8.245)	
Asian	-0.846 (-3.779–2.087)	
Native Hawaiian/Pacific Islander	6.686 (-4.722–18.09)	
Other Race	-3.814 (-9.815–2.187)	
Unknown	-4.133 (-14.21–5.944)	
**Annual Household Income**		
>=$150k	Reference	
0-<$50k	-9.618 (-15.46 - -3.771)	*p* < 0.01
2, $50-<$100k	-1.599 (-5.788–2.590)	
100k-<150k	2.557 (-1.041–6.156)	
declined/don’t know/missing	-1.546 (-5.529–2.437)	
**Education Level**		
No degree	Reference	
Associate’s degree	3.285 (-3.841–10.41)	
Bachelor’s Degree	1.488 (-3.701–6.677)	
Master’s Degree	3.587 (-1.780–8.955)	
professional/doctorate	5.233 (-0.838–11.30)	*p* < 0.1
**Gender**		
Male	Reference	
Female	1.288 (-1.263–3.840)	
**County**		
Santa Clara County	Reference	
Alameda County	2.083 (-1.529–5.694)	
Contra Costa County	1.766 (-2.694–6.226)	
Marin County	1.477 (-3.507–6.461)	
San Francisco County	2.934 (-1.375–7.244)	
San Mateo County	2.602 (-1.202–6.406)	

## Discussion

To our knowledge, this is the first study using the VHS to assess pediatric COVID-19 vaccine hesitancy in the Bay Area. More importantly, our study is done after FDA authorization of bivalent COVID-19 for children as young as 6 months. The strength of the VHS lies in its ability to identify vaccine-hesitant parents regardless of children’s vaccination status. This allows us to characterize not only current delays in vaccine uptake but also attitudes toward subsequent vaccination series, both of which are difficult to assess with vaccination status alone. For example, while most participants in our study sample consisted of highly educated parents with an uptake of the first dose of COVID-19 vaccine as high as 98.4%, only 87.3% of their index children received the first dose. This suggests that even among parents who are generally accepting vaccination, there is delay or refusal, defined by the WHO as hesitancy [15]. The VHS allows for the identification of these parents, to allow for an exploration of the complex factors underlying their hesitancy. Nonetheless, children’s vaccination status remains an actionable measure of vaccine uptake, especially for clinicians. It is therefore important that any tool assessing parents’ attitudes toward vaccination also reflects children’s vaccination status. Our CoV-VHS showed that parents who vaccinated their children were more likely to be vaccine-accepting, thereby strengthening the internal validity of the survey tool. The CoV-VHS should therefore be considered when assessing vaccine hesitancy in other populations to inform appropriate public health interventions.

Additionally, our results suggest that lower-income parents (lower than $50,000) in the Bay Area appear to be more vaccine-hesitant than higher-income parents ($150,000 or greater). This finding is consistent with previous national and city-specific studies assessing the influence of demographic factors on hesitancy [4, 9–11]. Interestingly, racial and ethnic group membership did not emerge as significant factors in our multivariate linear analyses. Similarly, there was no significant association between VHS score and parents’ county of residence, gender, age, or highest level of education. Our descriptive analysis of parental attitudes toward vaccination in terms of contextual, individual/group and vaccine-specific influences suggest the following: compared to parents who are less vaccine hesitant, those who are more vaccine hesitant believe that the COVID-19 vaccine is not needed for their child and do not trust the government is making decision in the best interest of their health (supplemental Fig. 1). These findings invite for a qualitative assessment of reasons for vaccine hesitancy in this population. Overall, our study underscores the need for public health interventions targeting vaccine hesitancy among lower-income parents.

Interventions to increase vaccine uptake are most effective when a multicomponent approach is tailored to the target population [21]. As a result, it is imperative that future research focuses on identifying the factors contributing to higher vaccine hesitancy in our population. After understanding these factors, interventions should include dialogue-based, incentive-based, and reminder/recall-based strategies [21].

Successful dialogue-based interventions have incorporated social mobilization, social media, mass media, and communication or information-based tools for healthcare workers. An example of a successful social mobilization campaign is presented in a 2009 study by Andersson et al. [22] Here, a cluster randomized controlled trial assessed the effect of informed discussion of vaccination costs and benefits on measles and diphtheria–pertussis–tetanus (DTP) vaccine uptake among children aged 12–23 months in poor districts in Pakistan’s Balochistan province. Intervention clusters had significantly higher vaccine uptake with a doubled odds ratio of measles vaccination (OR 2.20, 95% CI 1.24–3.88) and a triple odds ratio of DTP vaccination (OR 3.36, 95% CI 2.03–5.56) compared with control clusters. Social mobilization in the form of a vaccination cost/benefit analysis was therefore effective in this low-income population.

Incentive-based interventions, such as those addressing the basic needs of parents in low-income settings, have shown encouraging positive results. In a clustered randomized controlled trial in rural Rajasthan, India, Banerjee et al. [23] showed that small incentives addressing populations’ basic needs (raw lentils and metal plates in this case) have large positive impacts on the vaccine uptake, with a relative risk of completing immunization of 6.7 (4.5 to 8.8) for intervention groups versus control groups, in children aged 1–3 years. Reminder-recall interventions have also shown moderate effects in the case of DTP in low-income settings [24]. These strategies, however, appeared most efficient when used in tandem with other interventions [21]. Ultimately, the best interventions have been found to be context-dependent and require a deep understanding of the specific barriers to vaccine hesitancy in low-income populations.

In the context of the California Bay area, a multimodal approach that focuses on improving access to vaccination sites, health communication with the population, and relationships with community members might be effective. Concretely, public health departments could conduct individual interviews and organize focus group discussions with low-income parents in counties with the lowest vaccination rates to identify reasons for hesitancy and appropriate incentives to use. Additionally, public health officials and health care providers might use mobile vaccination clinics to circumvent any accessibility issues. Another recommendation is to provide health care professionals with communication training focused on addressing concerns and uncertainties about COVID-19 vaccination, including for patients who do not speak English and those with low health literacy.

### Limitations

The sample size of 251 participants limits the external validity of the study. Additionally, the racial and ethnic distribution of our sample does not adequately reflect that of the national population. Similarly, the longitudinal TrackCOVID study imposes its selection bias on our nested study. Participants of the TrackCOVID study, with Stanford and UCSF as study sites, are likely more health-conscious and inclined to vaccination than the national population. Further, through convenience sampling, only 27.6% TrackCOVID participants completed the screening survey of our study. This might have overrepresented participants who are more health-conscious at the expense of those who are not. These factors impede the generalizability of the study’s results at the national scale. Therefore, our findings might remain most applicable to the Bay Area population, but the CoV-VHS remains useful for studies in other local and national populations. Temporality and causal relationships are unable to be established due to the cross-sectional design of our study. Future work could follow populations through a serial cross-sectional design, or through a longitudinal cohort study to assess how attitudes change over the course of the public health concern [25, 26]. Additionally, no single scale can capture the complex and context-dependent factors contributing to vaccine hesitancy. While we performed a descriptive analysis of the influences of vaccine hesitancy in this population, a quantitative approach did not allow to collect nuanced information about these influences. Therefore, a qualitative assessment of the complex reasons underlying vaccine hesitancy in this population through individual interviews and focus groups might be required. Despite these limitations, our study reveals socioeconomic trends that can inform future investigation and public health interventions to increase vaccination and decrease hesitancy.

### Public health implications

Overall, this study provides the CoV-VHS, an adaptation of the VHS which allows for a standardized identification of vaccine-hesitant individuals in the setting of COVID-19. We recommend the use of the CoV-VHS in assessing vaccine hesitancy in other populations. More importantly, we report that low-income parents (annual income lower than $50,000) appear to have higher vaccine hesitancy compared to high-income parents (annual income greater than $150,000) in the Bay Area. This trend in income and vaccine hesitancy has been reported in other populations and further highlights socioeconomic disparities in our COVID-19 response. Future work and public interventions should focus on low-income parents. An important next step is to initiate an informed dialogue with parents through a qualitative assessment of the reasons contributing to higher vaccine hesitancy in this population with key informant interviews and focus groups. This qualitative assessment could also explore appropriate incentives that can be provided by public health officials. Additionally, dialogue-building skills can be reinforced among health care providers through interpersonal training and workshops inclusive of non-English speaking parents. Another recommendation is to deploy mobile vaccination clinics to improve accessibility for low-income individuals who might find them useful. Increasing vaccination rates for a preventable disease that disproportionally affects the most financially vulnerable individuals requires listening to their voices and addressing their concerns equitably.

## Electronic supplementary material

Below is the link to the electronic supplementary material.

Supplementary Material 1

## Data Availability

The data that support the findings of this study are not publicly available. The data are, however, available from the authors upon reasonable request and with the permission of Stanford University School of Medicine.

## Appendix

### COVID-19 VHS questionnaire

- The COVID-19 vaccine is important for my child’s health.
- The COVID-19 vaccine has NOT been around long enough to be sure that it’s safe.
- Getting the COVID-19 vaccine is a good way to protect my child from COVID-19.
- The COVID-19 vaccine is effective.
- I am concerned about serious side effects of the COVID-19 vaccine.
- Having my child get the COVID-19 vaccine is important for the health of others in my community.
- The COVID-19 vaccine is beneficial for my child.
- I think the COVID-19 vaccine might cause lasting health problems for my child.
- I do/did what my child’s health care provider recommends about the COVID-19 vaccine.
