# Supplementary material for: Low parental income level is associated with pediatric COVID-19 vaccine hesitancy in the San Francisco Bay area
Source: BMC Public Health. 2025 Mar 7;25:921. doi: 10.1186/s12889-025-22132-5 (PMC11889740; doi:10.1186/s12889-025-22132-5)
Supplement: Supplementary file 1 — Supplementary Material 1 [file 12889_2025_22132_MOESM1_ESM.docx]

**SUPPLEMENTAL MATERIAL:**

**Supplemental figure 1: Diverging Stacked Bar Chart of Influences of Vaccine Hesitancy in Vaccine Hesitant parents and Non-Vaccine Hesitant parents**

**
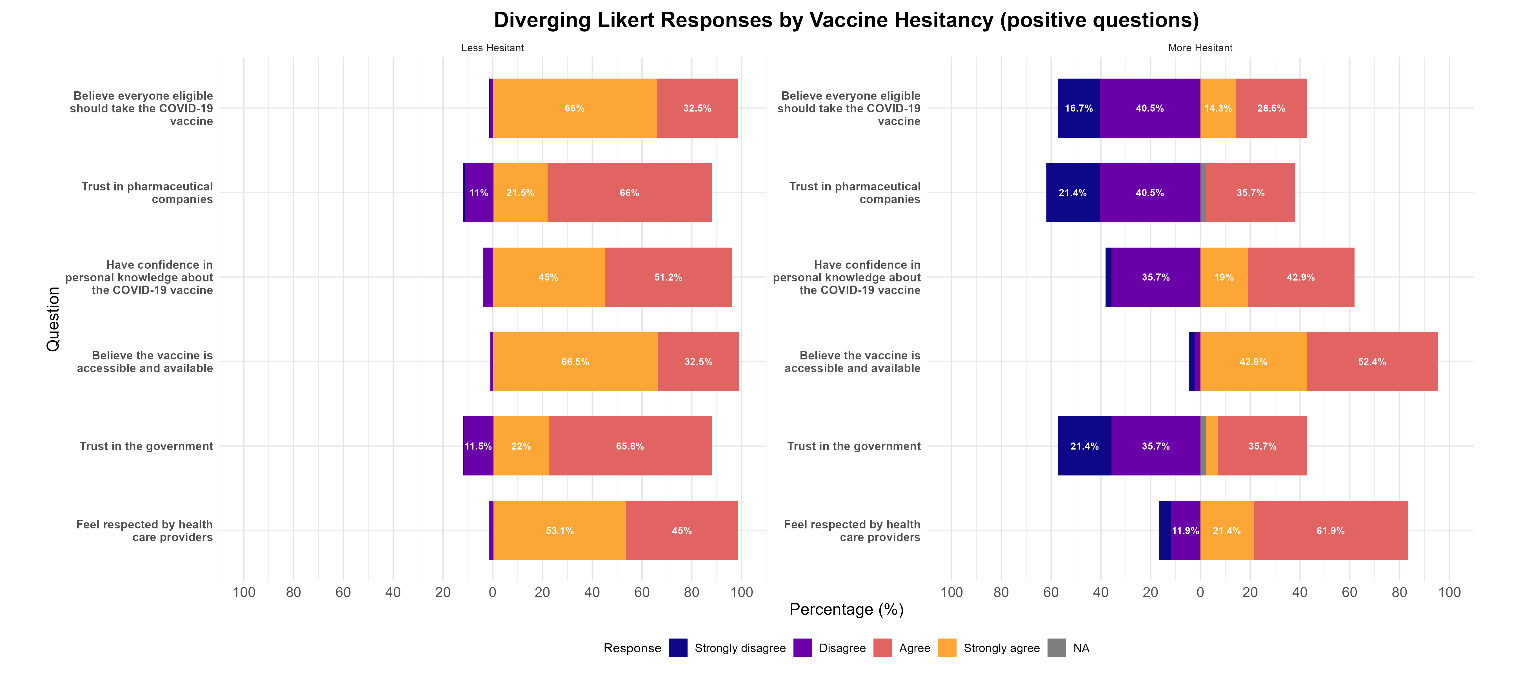
**

**
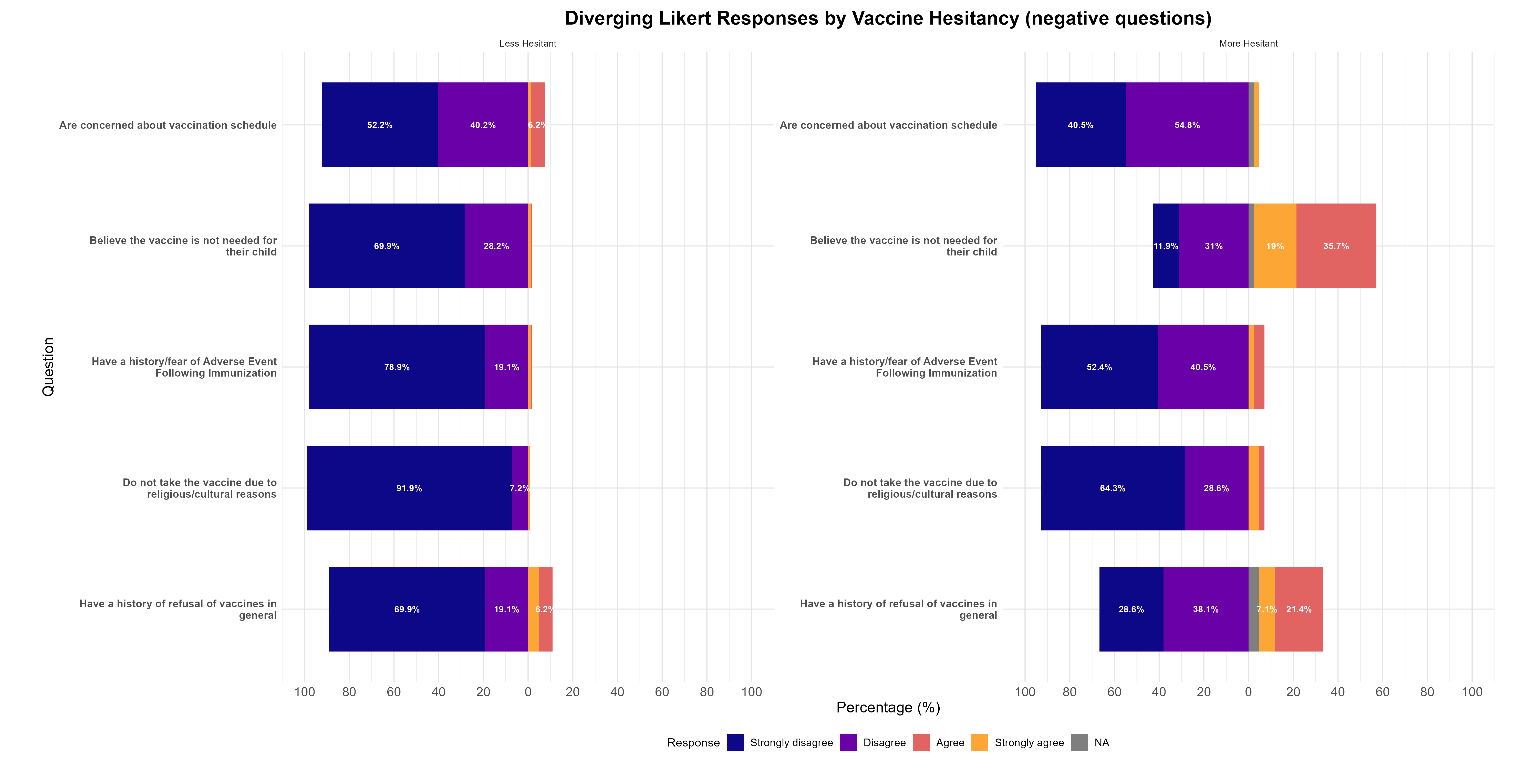
**
